# The Effect of Trunk Stability Training Based on Visual Feedback on Trunk Stability, Balance, and Upper Limb Function in Stroke Patients: A Randomized Control Trial

**DOI:** 10.3390/healthcare9050532

**Published:** 2021-05-02

**Authors:** Seok-Hui Yang, Eun-Jung Chung, Jin Lee, Su-Hyun Lee, Byoung-Hee Lee

**Affiliations:** 1 Graduate School of Physical Therapy, Sahmyook University, Seoul 01795, Korea; cuki1226@hanmail.net; 2 Department of Physical Therapy, Andong Science College, Andong 36616, Korea; qkskskzl@nate.com; 3 Department of Physical Therapy, Sahmyook University, Seoul 01795, Korea; leejin87@hanmail.net (J.L.); suhyunlee0811@gmail.com (S.-H.L.)

**Keywords:** visual feedback, trunk stability, upper limb function

## Abstract

This study aimed to investigate the effects of trunk stability training based on visual feedback on trunk stability, balance, and upper limb function in patients with stroke. Twenty-eight patients with chronic stroke were randomly assigned to either a trunk support group (*n* = 14) or a trunk restraint group (*n* = 14) that practiced upper limb training with trunk support and trunk restraint, respectively, based on visual feedback for 30 min per day, three times per week, for 4 weeks. The postural assessment scale for stroke (PASS) was used to assess the stability of patients, and the functional reaching test (FRT) was performed to assess balance. To assess upper extremity function, a range of motion (ROM) test, manual muscle testing (MMT), and Fugl–Meyer assessment-upper limb (FMA-upper limb) were performed. Consequently, both groups showed significant differences before and after training in the PASS, FRT, shoulder flexion ROM, triceps brachii MMT, and FMA-upper limb (*p* < 0.05), while the trunk support group showed more significant improvements than the trunk restraint group in the PASS, FRT, and FMA-upper limb (*p* < 0.05). Trunk support-based upper limb training effectively improved trunk stability, balance, and upper limb function and is beneficial as an upper limb training method. Providing trunk support is more effective than restricting the trunk; trunk support-based upper limb training is expected to promote voluntary participation when combined with visual feedback.

## 1. Introduction

Approximately 66% of patients with stroke suffer from impairments in daily life due to impaired function and movement of the upper limbs [1]. Regaining the optimal level of use of the damaged upper limbs is important, as upper limb function is essential for daily life activities [2]. Although strength exercises [3], task-oriented practice [4], and upper limb training using robots [5] have been used to address upper limb function problems in patients with stroke, these interventions do not provide the patient with instantaneous corrective information about movement errors during training [6]. Visual feedback-based upper extremity training is often used as a method for correcting movement errors [7].

Among various intervention methods, visual feedback-based upper extremity training can help improve balance and induce interest in rehabilitation in patients with stroke [8]. During visual feedback, the mirror neurons are activated when we observe behavior, resulting in forming a new neural pathway in the primary motor cortex when following the motion [9]. Therefore, if the information is provided using visual feedback when performing a movement, it may benefit the patients [8]. However, if good information is provided and the patient cannot accommodate it, the performance will be adversely affected [6]. Patients with stroke have a markedly low level of intrinsic feedback during exercise; therefore, if excessive external feedback is provided, they may use excessive and unnecessary movement when performing the action. Therefore, patients with a low capacity to accommodate feedback effect should be provided with a device to support their capacity [9].

When the function of the upper limbs of stroke patients decreases, it becomes difficult to perform movements to reach the target [10]. For the upper extremity to reach the target, the shoulder and elbow should be stretched to increase the length of the arm’s leverage, and the center of gravity of the arm should be dislocated from the center of gravity of the body [11]. To achieve this, trunk stability is needed for maintaining balance and posture while moving the upper extremity [12]. However, stroke patients have reduced trunk stability, resulting in the upper extremity being unable to reach the target, compensation for flexion, rotation of the trunk to compensate for insufficient distances, and reduced circumstances in which the shoulders and elbows can vary in length [13]. Decreased trunk stability in stroke patients is also a problem when performing upper extremity training with visual feedback [14]. This indicates that the intrinsic feedback ability to learn exercise is low and methods should be presented to aid the body's stability when applying visual feedback [6].

Since the lack of trunk stability is one of the representative problems of patients with stroke, many previous studies have focused on training the upper body using restraint [10] or support [11]. Both methods are intended to palliate the compensatory actions that occur during the reaching motion as the system collapses after a stroke [10,11]. In previous studies that assessed intervention methods, inter-body restraint-based training had a significant effect on active shoulder flexion and inter-body displacement reduction in the reaching exercise; however, it did not show a significant difference concerning upper limb function [10]. In one study, training using trunk support was found to improve the functioning of the proximal region as opposed to that of the upper extremity, but quantifying the improvement in the upper extremity function test was challenging [11]. Both types of interventions affected trunk stability, but there was no significant difference in their effect on upper limb function. Moreover, it is insufficient to provide the ability to substantially improve upper extremity function. In addition, visual feedback is useful for inducing voluntary participation [8].

Therefore, this study aimed to investigate the effects of trunk stability training based on visual feedback on trunk stability, balance, and upper limb function in patients with stroke. Additionally, to determine an effective method of providing trunk stability, we compared the differences between trunk support-based and trunk restraint-based upper limb training.

## 2. Materials and Methods

### 2.1. Subjects

We initially selected 32 patients diagnosed with chronic stroke and admitted to a rehabilitation hospital in Seoul. They all signed a consent form after the procedure and purpose of the study were explained. The selection criteria were as follows: onset of stroke 6 months before the study, able to sit on a wheelchair for more than 30 min, able to follow simple instructions of the researcher (Korean version of mini-mental state exam 21 scores or more), and a modified Ashworth scale (MAS) score ≤2 to minimize the possibility of natural recovery. The exclusion criteria were as follows: the presence of an orthopedic disease affecting the cardiovascular system and upper extremities, visual impairments and visual field defects, and recent participation in similar studies. Participation could be withdrawn at any time during the study. This study was conducted with the approval of the Research Institutional Review Board of Sahmyook University (approval number: 2–7001793-AB-N-012018022HR). The objectives and procedures used in the study were fully understood by the subjects. This study upheld the ethical principles of the Declaration of Helsinki.

### 2.2. Experimental Procedure

Before recruiting participants for this study, we performed a power analysis using G*Power version 3.1.9.7 (Heinrich-Heine Universität, Düsseldorf, Germany); an overall effect size index of 0.53 was obtained for all the outcome measures, with a probability of 0.05, to minimize type II errors (power of 80%). Because the estimated target sample size was 30, we recruited 32 participants undergoing rehabilitation and physical therapy after a stroke. After the screening test, two patients were excluded: one with a Korean mini-mental state examination (K-MMSE) score <21 and another with a low condition. The stability, balance, and upper limb function of the trunk were measured by a preliminary test. The 30 participants were randomly divided into two groups: the trunk support-based (*n* = 15) and trunk restraint-based upper limb training (*n* = 15) groups. Both groups performed training for 30 min a day, three times a week, for 4 weeks.

**Figure 1:**
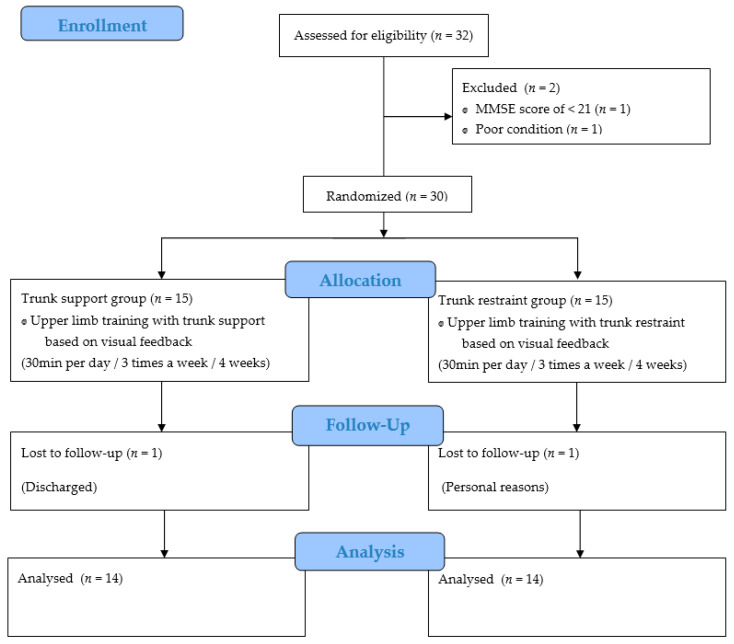
Flow diagram of the overall experimental procedure.

Those who were unable to participate in the experiment or whose participation rate was <80% because of discharge and personal reasons were excluded from the final study. In the trunk support group, one discharged patient discontinued, and in the trunk restraint group, one patient discontinued for personal reasons; thus, 14 patients participated in each group. Therefore, 28 participants were included in the final study. After completing the experiment, trunk stability, balance, and upper extremity function were measured in both groups using the same measurement tools as in the pre-test (Figure 1).

### 2.3. Training Program

This study was conducted by a physical therapist with more than 3 years of experience, and a training program was designed by the same therapist. To minimize errors that may occur in the experimental process, the patients were educated and allowed to familiarize themselves with the tools, measurement method, and training program before starting the experiment.

Both groups underwent sensory-motor active rehabilitation training (SMART) of the arm [15] with real-time feedback on upper limb exercise, provided using a camera. For real-time feedback, the participants sat on chairs and watched a 27-inch screen installed 2 m in front of them. A motion-observation screen and a real-time feedback screen were displayed on one monitor simultaneously at the participants’ eye level. The participants exercised in a sitting position, and the chair height was adjusted according to the knee flexed at 100° [16]. A webcam was placed in front of the participants so that they could see the feedback in real time. The experimenter took pictures of the participants using a camera and checked the appearance on the monitor to correct the posture. A total of 15 sets were performed on the sagittal plane, 10 times, with the affected upper limb reaching out for 1 min. One minute of rest after one set was allowed to minimize the occurrence of pain due to repetitive movements and to minimize the participation of the lower extremities to compensate for the excessive use of the upper extremity muscles. Before training the subjects, verbal directive cues were given, such as “Reach out your arm according to the movement observed on the screen” and “Follow the instructions as you modify the way you reach out your arm”. The experiment was conducted 30 times a day, three times a week, for 4 weeks.

**Figure 2:**
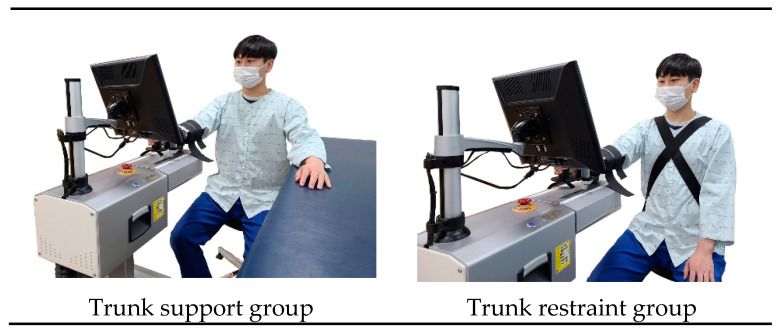
Sensory-motor active rehabilitation training of the arm.

In the trunk support group, upper limb support was provided using a table. The height of the table was adjusted to the patient’s shoulder flexion at 90° after confirming that the shoulder joint was not restricted. In the trunk restraint group, the patients were immobilized using restraint straps after confirming that the shoulder joint was not restricted (Figure 2).

### 2.4. Outcome Measures

Trunk stability tests were performed using the postural assessment scale for stroke (PASS). The PASS is a tool used for evaluating the ability of patients with stroke to perform posture control using the Fugl–Meyer balance scale. In addition, it is a useful clinical tool for diagnosing the patient’s condition, as it can be evaluated simply by making evaluation items difficult to apply to patients with posture control disorders. The test–retest reliability of the evaluation tool was good with an intraclass correlation coefficient (ICC) of 0.88–0.98, and the interrater reliability was good with an ICC of 0.77–0.99 [17]. The stroke posture evaluation scale included three basic postures, lying, sitting, and standing, using a total of 12 items comprising of five items for posture maintenance and seven items for posture change; a minimum of 0 to a maximum of 3 points could be earned for each item, resulting in a total of 36 points.

The functional reaching test (FRT) was conducted to evaluate the patients’ balance. The FRT evaluates the limit of stability by measuring the maximum distance an individual can reach forward while standing in a fixed position. The patients were instructed to stand with their feet shoulder-distance apart, make a fist, and raise their arms up. After recording the first point of the distal end of the 3rd metacarpal, the level of the bar was maintained horizontally at the height of the shoulders in the starting position while maintaining the level of the upper extremity for 5 s without losing balance. By measuring the difference in the distance between the first and last points, the balance limits were measured appropriately. When the FRT results were parametrized and analyzed, the retest reliability and inter-test reliability were 0.987 and 0.983, respectively [18]. In this study, for convenience of measurement, a scale was drawn and marked on the treatment room wall, and the average value was obtained from three repeated measurements.

Upper limb function on the affected side was determined by the range of motion (ROM) of the shoulder flexion joint, manual muscle test (MMT) of the triceps muscle, and Fugl–Meyer assessment-upper limb (FMA-upper limb). The ROM of shoulder joint flexion was recorded during movement. The test–retest reliability and inter-measurement reliability were good with ICCs of 0.83 and 0.74, respectively [19]. Measurements were made using a general goniometer, and motion accuracy was ensured by performing measurements three times each, by the same examiner, and using the range in which the patients could move by themselves. Therefore, the effect of changes in shoulder flexion on the stretching motion was also reported.

The MMT is a six-point scale (0–5). It consists of a standardized test sequence, instructions for performing the test, and scores for each muscle. The test–retest reliability and inter-measurement reliability were good with ICCs of 0.96 and 0.93, respectively [20]. The motion was measured three times to obtain an average value, and both pre- and post-measurements were performed by the same examiner. For the hand to reach the target, stretching motion of the elbow joint is required, and the strength of the upper forearm muscle plays an important role in this regard [15]. Therefore, in this study, we also examined the effect of stretching motion by observing the changes in the strength of the triceps muscle.

The perfect score for the FMA is 100; the upper limbs are subdivided into the shoulder/elbow, wrist, and hand (finger), and the perfect score for the upper limbs is 66; the lower limbs are subdivided into the hip, knee, and ankle, and the perfect score for the lower limbs is 34. The reliability of the upper limb assessment was good, with an ICC of 0.99 [21]. The items of this evaluation scale were classified into three sections: 0 points indicating no upper limb function, 1 point indicating partial function, and 2 points indicating complete function.

### 2.5. Data Analysis

Data analysis was performed using SPSS ver. 19.0 (SPSS Inc., Chicago, IL, USA). Data were presented as mean and standard deviation. According to the results of the Shapiro–Wilk normality test, all items were normally distributed. The participants’ general characteristics were presented as descriptive statistics. Independent *t*-tests were used to compare the differences between the groups. A paired *t*-test was used to compare within-group means. The significance level for all tests was set at 0.05.

## 3. Results

Table 1 shows the general characteristics and homogeneity test results of the study subjects. There was no significant difference between the groups.

### 3.1. Trunk Stability

The groups showed a significant difference between before and after training in the PASS scores (*p* < 0.05), and there was a significant difference between pre-test and post-test scores in the trunk support group than that in the trunk restraint group (*p* < 0.01) (Table 2).

### 3.2. Balance

The groups showed a significant difference between before and after training in the FRT results (*p* < 0.001), and there was a significant difference between pre-test and post-test scores in the trunk support group compared to that in the trunk restraint group (*p* < 0.01) (Table 3).

### 3.3. Upper Limb Function

The groups showed a significant difference between before and after training in the ROM and MMT and FMA-upper limb scores (*p* < 0.05), and the trunk support group showed more significant differences than the trunk restraint group in the FMA-upper limb scores (*p* < 0.05) (Table 4).

## 4. Discussion

### 4.1. Trunk Stability

In this study, a stroke posture scale was used to evaluate the changes in trunk stability. The scores had increased by 2.71 points and 1.29 points in the trunk support and trunk restraint groups, respectively. There was a statistically significant difference between the groups (*p* < 0.05). Dai et al. [22] reported that the mean balancing exercise score had increased from 12.88 points to 19.29 points with virtual rehabilitation and trunk stability-based upper limb training, showing a significant difference. Wee et al. [10] reported that when the upper limb reaches an object within its length, the shoulders and elbows stretch, and the center of gravity of the arm deviates from the center of gravity of the body. Therefore, to maintain posture, the trunk should be able to act as a device that maintains stability while moving the arm [10]. Patients with stroke are unable to maintain trunk stability and cannot prepare the body for disturbance to the outside; however, both groups achieved trunk stability that assisted in maintaining posture and adapting to complex visual feedback.

### 4.2. Balance

In this study, a functional upper limb stretch test and stroke posture scale were used to assess the changes in balance. In the functional upper limb stretch test, the scores of the trunk support and trunk restraint groups increased significantly from 14.80 cm to 16.88 cm after the experiment and from 14.86 cm to 16.00 cm before the experiment, respectively. When comparing the before and after training, the trunk support group showed a significant increase (*p* < 0.05). Kalron et al. [23] used the functional stretch test to evaluate the improvement in balance with virtual rehabilitation and reported a significant increase from 30.4 cm to 34.8 cm. Aruin et al. [24] concluded that one must adapt to environmental changes to maintain balance. It is important to have the physical ability to maintain balance while moving. Kalron et al. [23] reported that the key factor in maintaining balance is proper training according to the individual’s abilities. It is assumed that stability helped control the excessive movement due to the visual feedback. If a patient experiences improved function, it would lead to excessive movement, and the balancing ability may decrease as the posture collapses.

### 4.3. Upper Limb Function

In this study, we attempted to increase the patients’ participation through visual feedback and to determine how this method affected upper limb function. Bets et al. [25] analyzed shoulder joint flexion in patients with stroke. In bending angles ranging from 60° to 120°, the serratus anterior muscle was activated slowly as the lower trapezius and infraspinatus muscles responded slowly, affecting the movement of the shoulder joint and chest. If trunk stability is provided, inaccurate movements of the trunk are reduced, and the visual feedback can enable self-correction, which can increase the ROM of the joint. In this study, the ROM of the trunk support group increased by 2.86°, and that of the trunk restraint group increased by 2.07°. The range of joint movements increased to more than the angle of movement in the exercise program, and the shoulder joint movement range increased through repetitive movements, thus increasing the angle of anterior flexion.

The strength of the triceps muscle plays an important role in reaching the target position when performing stretch movements [15]. When the movement of the trunk is improved by providing trunk stability during the same movement repetitively, the movement will be smooth, causing the strengthening of the triceps. In this study, the strength of the triceps muscle increased by 0.46 points in the trunk support group and by 0.25 points in the trunk restraint group. This suggests that the frequency of muscle use increases as the burden on the trunk decreases, thus improving muscle strength.

In stroke patients, asymmetric activation of trunk muscles reduces the stability of the body, and the decrease in trunk stability is largely associated with a decrease in balancing ability [26]. The decrease in trunk stability makes it difficult to maintain posture and balance, which results in postural sway [27]. This trunk sway increases the incidence of falls in stroke patients, as well as the fear of falls [28]. Stroke patients may experience abnormal muscle tone to prevent trunk sway [29]. Muscle synergies have been identified as components for various motor tasks in humans, including postural responses [30], locomotion [31], hand shaping [32], isometric force generation in the upper extremity [33], and reaching movements performed under different biomechanical constraints [34]. Specifically, stroke is associated with the emergence of particularly strong coupling of elbow flexion and shoulder antigravity torques and posture-dependent coupling between elbow extension and shoulder adduction torques [35]. An increase in abnormal muscle tone in the body can increase the flexor synergy pattern in stroke patients [36]. Thus, an increased flexor synergy pattern in the upper extremity due to reduced trunk stability reduces upper extremity function in stroke patients [10].

The FMA-upper limb provides an objective and comprehensive evaluation of the movement components of the upper limbs [21]. In this study, the FMA-upper limb score increased statistically by four points in the trunk support group and by 1.36 points in the trunk restraint group. There was a statistically significant difference between the groups (*p* < 0.05). Visual feedback induces spontaneous participation in upper limb function and is assumed to have had a positive effect as the number of movements increased. Houwink et al. [37] reported that tasks are performed smoothly if attention is reduced when attempting to perform them. This suggests that when the upper extremity is supported, the attention deployed to the affected area is reduced, and this seems to have a positive effect as the complexity of the exercise reduces when the upper extremity is supported against gravity.

This study has a number of limitations. First, there was a potential lack of statistical power due to the small sample size. Therefore, our results cannot be generalized to the entire stroke population. Future research should be performed using a larger sample size. Second, we could not obtain follow-up effects of trunk stability training based on visual feedback. Future work will examine clinical outcomes in a follow-up study. Third, measuring changes in the timing of muscle activation for posture control can help determine the changes in exercise learning [21]. In this study, muscle movement could not be analyzed. These data should be analyzed, and future studies could examine the features of various movements.

## 5. Conclusions

We found that upper limb training with trunk support and visual feedback supported trunk stability, balance, and upper limb function. Improvements in trunk stability and upper limb function were confirmed when the trunk support and trunk restraint groups were compared. In this study, stability was provided using a desk, whereas restraint was achieved by restraining the entire trunk using straps. Although restraining the entire trunk eliminates unnecessary movement of the trunk, it can hinder the movement of the affected side, thus preventing the upper limbs from moving in sync with the scapula and trunk. Restraining the entire trunk prevents movement and limits the upper limbs. Based on the results of this study, the support method was better than the restraint method in improving the stability, balance, and upper extremity functions of the trunk. Trunk support training can help improve voluntary participation with the help of visual feedback.

## Figures and Tables

**Table 1:** General characteristics of participants (*N* = 28).

Characteristics	Trunk Support Group (*n* = 14)	Trunk Restraint Group (*n* = 14)	t	*p*/x^2^
Sex (male/female)	12/2	12/2	0.000	1.000
Ages (years)	62.64 ± 9.24	65.93 ± 11.96	−0.813	0.579
Height (cm)	165.29 ± 7.46	170.93 ± 7.95	−1.935	0.946
Weight (kg)	64.71 ± 9.94	65.86 ± 11.41	−0.283	0.611
Affected side (left/right)	8/6	8/6	0.000	1.000
Post-stroke duration (months)	30.57 ± 15.47	29.43 ± 17.81	0.181	0.413
Body mass index	23.57 ± 2.25	22.36 ± 2.65	1.305	0.674
K-MMSE (score)	25.57 ± 3.54	26.50 ± 3.05	−0.742	0.626

Values are expressed as mean ± standard deviation; K-MMSE = Korean mini-mental state examination.

**Table 2:** Comparison of trunk stability (*N* = 28).

Trunk Stability	Trunk Support Group (*n* = 14)	Trunk Restraint Group (*n* = 14)	t	*p*/x^2^
PASS (score)				
Pre-test	25.29 ± 6.37	26.07 ± 4.74		
Post-test	28.00 ± 5.60	27.36 ± 4.39		
Pre-post	2.71 ± 1.20	1.29 ± 1.06	3.319	0.003
*t* (*p*)	−8.432 (0.00)	−4.500 (0.01)		

Values are expressed as mean ± standard deviation; PASS = postural assessment scale for stroke.

**Table 3:** Comparison of balance (*N* = 28).

Balance	Trunk Support Group (*n* = 14)	Trunk Restraint Group (*n* = 14)	t	*p*/x^2^
FRT (score)				
Pre-test	14.80 ± 4.18	14.86 ± 3.85		
Post-test	16.88 ± 4.00	16.00 ± 3.80		
Pre-post	2.07 ± 0.83	1.13 ± 0.65	3.327	0.003
*t* (*p*)	−9.356 (0.000)	−6.456 (0.000)		

Values are expressed as mean ± standard deviation; FRT = functional reaching test.

**Table 4:** Comparison of upper limb function (*N* = 28).

Upper Limb Function	Trunk Support Group (*n* = 14)	Trunk Restraint Group (*n* = 14)	t	*p*/x^2^
ROM (°) (shoulder flexion)				
Pre-test	139.29 ± 25.50	138.21 ± 23.10		
Post-test	142.14 ± 24.59	140.29 ± 21.19		
Pre-post	2.86 ± 2.28	2.07 ± 2.12	0.942	0.355
*t* (*p*)	−4.684 (0.000)	−3.640 (0.03)		
MMT (score) (triceps brachii)				
Pre-test	3.21 ± 1.29	3.46 ± 1.08		
Post-test	3.67 ± 0.84	3.71 ± 0.91		
Pre-post	0.46 ± 0.57	0.25 ± 0.32	1.221	0.233
*t* (*p*)	−3.045 (0.009)	−3.640 (0.03)		
FMA-upper limb (score)				
Pre-test	40.71 ± 12.95	44.57 ± 12.10		
Post-test	44.71 ± 12.05	45.93 ± 11.44		
Pre-post	4.00 ± 1.79	1.36 ± 1.21	4.577	0.000
*t* (*p*)	−8.327 (0.00)	−4.177 (0.01)		

Values are expressed as mean ± standard deviation; ROM = range of motion; MMT = manual muscle test; FMA-upper limb = Fugl–Meyer assessment-upper limb test.

## Data Availability

Not applicable.

## References

[1] Dobkin B.H. (2005). Rehabilitation after stroke. N. Engl. J. Med..

[2] Franck J.A., Smeets R., Seelen H.A.M. (2017). Changes in arm-hand function and arm-hand skill performance in patients after stroke during and after rehabilitation. PLoS ONE.

[3] Graef P., Michaelsen S.M., Dadalt M.L.R., Rodrigues D.A.M.S., Pereira F., Pagnussat A.S. (2016). Effects of functional and analytical strength training on upper-extremity activity after stroke: A randomized controlled trial. Braz. J. Phys..

[4] Rensink M., Schuurmans M., Lindeman E., Hafsteinsdóttir T. (2009). Task-oriented training in rehabilitation after stroke: Systematic review. J. Adv. Nurs..

[5] Veerbeek J.M., Langbroek-Amersfoort A.C., Wegen E.E.H., Meskers C.G.M., Kwakkel G. (2017). Effects of Robot-assisted therapy for the upper limb after stroke. Neurorehabil. Neural Repair.

[6] Lauber B., Keller M. (2014). Improving motor performance: Selected aspects of augmented feedback in exercise and health. Eur. J. Sport Sci..

[7] Brunner I., Skouen J.S., Hofstad H., Aßmuss J., Becker F., Pallesen H., Thijs L., Verheyden G. (2016). Is upper limb virtual reality training more intensive than conventional training for patients in the subacute phase after stroke? An analysis of treatment intensity and content. BMC Neurol..

[8] Shiri S., Feintuch U., Lorber-Haddad A., Moreh E., Twito D., Tuchner-Arieli M., Meiner Z. (2012). Novel virtual reality system integrating online self-face viewing and mirror visual feedback for stroke rehabilitation: Rationale and feasibility. Top Stroke Rehabil..

[9] Kumru H., Albu S., Pelayo R., Rothwell J., Opisso E., Leon D., Soler D., Tormos J.M. (2016). Motor cortex plasticity during unilateral finger movement with mirror visual feedback. Neural Plast..

[10] Wee S.K., Hughes A.M., Warner M., Burridge J.H. (2014). Trunk restraint to promote upper extremity recovery in stroke patients: A systematic review and meta-analysis. Neurorehabil. Neural Repair.

[11] Chan I.H., Fong K.N.K., Chan D.Y.L., Wang A.Q.L., Cheng E.K.N., Chau P.H.Y., Chow K.K.Y., Cheung H.K.Y. (2016). Effects of arm weight support training to promote recovery of upper limb function for subacute patients after stroke with different levels of arm impairments. Biomed. Res. Int..

[12] Kanekar N., Aruin A.S. (2014). Aging and balance control in response to external perturbations: Role of anticipatory and compensatory postural mechanisms. Age.

[13] Lima R.C., Nascimento L.R., Michaelsen S.M., Polese J.C., Pereira N.D., Teixeira-Salmela L.F. (2014). Influences of hand dominance on the maintenance of benefits after home-based modified constraint-induced movement therapy in individuals with stroke. Braz. J. Phys..

[14] Schrafl-Altermatt M., Dietz V. (2016). Neural coupling of cooperative hand movements after stroke: Role of ipsilateral afference. Ann. Clin. Transl. Neurol..

[15] Brauer S.G., Hayward K.S., Carson R.G., Cresswell A.G. (2013). Ruth N Barker The efficacy of SMART Arm training early after stroke for stroke survivors with severe upper limb disability: A protocol for a randomised controlled trial. BMC Neurol..

[16] Pereira S., Silva C.C., Ferreira S., Silva C., Oliveira N., Santos R., Vilas-Boas J.P., Correia M.V. (2014). Anticipatory postural adjustments during sitting reach movement in post-stroke subjects. J. Electromyogr. Kinesiol..

[17] Persson C.U., Hansson P.O., Danielsson A., Sunnerhagen K.S. (2011). A validation study using a modified version of Postural Assessment Scale for Stroke Patients: Postural Stroke Study in Gothenburg (POSTGOT). J. Neuroeng. Rehabil..

[18] Merchán-Baeza J.A., González-Sánchez M., Cuesta-Vargas A.I. (2014). Reliability in the parameterization of the functional reach test in elderly stroke patients: A pilot study. Biomed Res. Int..

[19] Kolber M.J., Vega F., Widmayer K., Cheng M.S.S. (2011). The reliability and minimal detectable change of shoulder mobility measurements using a digital inclinometer. Physiother Theory Pr..

[20] Pandian S., Arya K.N. (2013). Motor impairment of the ipsilesional body side in poststroke subjects. J. Bodyw. Mov..

[21] Pandian S., Arya K.N., Kumar D. (2015). Effect of motor training involving the less-affected side (MTLA) in post-stroke subjects: A pilot randomized controlled trial. Top Stroke Rehabil..

[22] Dai C.Y., Huang Y.H., Chou L.W., Wu S.C., Wang R.Y., Lin L.C. (2013). Effects of primary caregiver participation in vestibular rehabilitation for unilateral neglect patients with right hemispheric stroke: A randomized controlled trial. Neuropsychiatr. Dis. Treat.

[23] Kalron A., Fonkatz I., Frid L., Baransi H., Achiron A. (2016). The effect of balance training on postural control in people with multiple sclerosis using the CAREN virtual reality system: A pilot randomized controlled trial. J. Neuroeng. Rehabil..

[24] Aruin A.S. (2016). Enhancing Anticipatory Postural Adjustments: A Novel Approach to Balance Rehabilitation. J. Nov. Physiother.

[25] De Baets L., Deun S.V., Monari D., Jaspers E. (2016). Three-dimensional kinematics of the scapula and trunk, and associated scapular muscle timing in individuals with stroke. Hum. Mov. Sci..

[26] Haruyama K., Kawakami M., Otsuka T. (2017). Effect of core stability training on trunk function, standing balance, and mobility in stroke patients. Neurorehabil. Neural Repair.

[27] Cabanas-Valdés R., Bagur-Calafat C., Girabent-Farrés M., Caballero-Gómez F.M., Hernández-Valiño M., Cuchí G.U. (2016). The effect of additional core stability exercises on improving dynamic sitting balance and trunk control for subacute stroke patients: A randomized controlled trial. Clin. Rehabil..

[28] Lee N.G., You J.S.H., Yi C.H., Jeon H.S., Choi B.S., Lee D.R., Park J.M., Lee T.H., Ryu I.T., Yoon H.S. (2018). Best core stabilization for anticipatory postural adjustment and falls in hemiparetic stroke. Arch. Phys. Med. Rehabil..

[29] Merkert J., Nieczaj S.B.R., Steinhagen-Thiessen E., Eckardt R. (2011). Combined whole body vibration and balance training using Vibrosphere^®^: Improvement of trunk stability, muscle tone, and postural control in stroke patients during early geriatric rehabilitation. Z Gerontol. Geriatr..

[30] Torres-Oviedo G., Macpherson J.M., Ting L.H. (2006). Muscle synergy organization is robust across a variety of postural perturbations. J. Neurophysiol..

[31] Clark D.J., Ting L.H., Zajac F.E., Neptune R.R., Kautz S.A. (2010). Merging of healthy motor modules predicts reduced locomotor performance and muscle coordination complexity post-stroke. J. Neurophysiol..

[32] Ajiboye A.B., Weir R.F. (2009). Muscle synergies as a predictive framework for the EMG patterns of new hand postures. J. Neural Eng..

[33] Roh J., Rymer W.Z., Beer R.F. (2012). Robustness of muscle synergies underlying three-dimensional force generation at the hand in healthy humans. J. Neurophysiol..

[34] Muceli S., Boye A.T., d’Avella A., Farina D. (2010). Identifying representative synergy matrices for describing muscular activation patterns during multidirectional reaching in the horizontal plane. J. Neurophysiol..

[35] Ellis M.D., Acosta A.M., Yao J., Dewald J.P.A. (2007). Position-dependent torque coupling and associated muscle activation in the hemiparetic upper extremity. Exp. Brain Res..

[36] Roh J., Rymer W.Z., Perreault E.J., Yoo S.B., Beer R.F. (2013). Alterations in upper limb muscle synergy structure in chronic stroke survivors. J. Neurophysiol..

[37] Houwink A., Steenbergen B., Prange G.B., Buurke J.H., Geurts A.C.H. (2013). Upper-limb motor control in patients after stroke: Attentional demands and the potential beneficial effects of arm support. Hum. Mov. Sci..

